# A Narrative Review of Shockwave Therapy in Plantar Fasciitis

**DOI:** 10.3390/jfmk11010123

**Published:** 2026-03-17

**Authors:** Yunfeng Sun, Caterina Fede, Xiaoxiao Zhao, Federico Giordani, Hannes Müller-Ehrenberg, Carmelo Pirri, Carla Stecco

**Affiliations:** 1 Padova Neuroscience Center, University of Padova, 35122 Padova, Italy or syfengst@foxmail.com (Y.S.); xiaoxiao.zhao@studenti.unipd.it (X.Z.); 2 Institute of Human Anatomy, Department of Neuroscience, University of Padova, 35122 Padova, Italy; caterina.fede@unipd.it (C.F.); carmelo.pirri@unipd.it (C.P.); 3 Villa Rosa Rehabilitation Hospital, APSS, 38057 Trento, Italy; federico.giordi@gmail.com; 4 Private Clinic Orthopädische Privatpraxis, 48143 Münster, Germany; info@triggerpunktzentrum.de

**Keywords:** plantar fasciitis, ESWT, shockwave, plantar fascia, foot pain

## Abstract

This narrative review synthesizes evidence from 108 studies to provide the first comprehensive overview of extracorporeal shockwave therapy (ESWT) for plantar fasciitis across three key domains. First, assessment methodologies were evaluated, identifying 36 distinct tools classified into six categories, including pain (with the Visual Analog Scale being the most frequently used), function (most commonly the Foot Function Index), plantar fascia thickness, and other measures. Second, treatment protocols were analyzed, revealing commonly applied parameters of 2000 impulses per session and an energy flux density of 0.2 mJ/mm^2^ or 3.0 bar. Third, the comparative status of ESWT relative to other interventions was examined. Across 18 alternative treatments, corticosteroid injections, platelet-rich plasma (PRP), dextrose prolotherapy, laser therapy, and ultrasound were the most frequently compared modalities. ESWT and comparator interventions demonstrated differential advantages across specific outcomes; however, these findings cannot be directly translated into clinical recommendations, due to the limitations of the available evidence. By consolidating fragmented data, the present review clarifies the current research landscape and provides a foundational reference to support outcome evaluation and individualized treatment selection.

## 1. Introduction

Plantar fasciitis (PF)-related pain and its associated symptoms represent one of the leading causes of impaired activities of daily living and reduced physical function, with an estimated lifetime incidence of approximately 10% in the general population [1]. PF is characterized by heel pain, local swelling, impaired mobility, and, in chronic cases, recurrence and psychological distress associated with long-term pathology [2]. The condition is commonly attributed to inflammatory responses or degenerative changes within the plantar fascia, a connective tissue structure extending from the calcaneus to the toes [2]. Previous studies have categorized contributing factors into three major domains: neurological-, skeletal-, and soft-tissue-related causes [2,3]. Clinically, the hallmark symptom of PF is heel pain, typically most pronounced during the first steps taken in the morning or following prolonged periods of standing. Individuals with obesity, occupations requiring prolonged standing, and biomechanical factors such as excessive ankle dorsiflexion are at increased risk of developing PF [4], particularly among adults aged 45–64 years old [5].

Pain, the predominant symptom of PF, may persist for months or years; more than 80% of patients report pain lasting longer than one year after diagnosis [4]. Addressing the pain and the pain-related pathology is the primary therapeutic strategy for PF [6]. Extracorporeal shock wave therapy (ESWT) is a common conservative treatment due to its convenience, time efficiency, and few clinical contraindications [7]. ESWT is regarded as a therapeutic approach with regenerative potential, as the energy imparted to bodily tissues can stimulate the formation of new blood vessels, enhance protein synthesis, promote cell proliferation, and facilitate the breakdown of calcifications within musculoskeletal frameworks [8,9]. ESWT contributes to improving the cellular metabolism, regulating tissue recovery, and relieving inflammation in plantar fasciitis [9,10]. ESWT is used independently or with other conservative therapies, including manipulation, injection, ultrasound, exercise, taping, laser, and pharmacological therapies [11].

In clinical practice, considerable attention has been directed toward ESWT prescription parameters for PF, including the number of pulses and energy flux density. However, the existing literature is marked by substantial heterogeneity, primarily arising from variations in outcome assessment tools, shock wave parameters, and reported clinical endpoints. These issues have been highlighted in previous studies [12] and continue to impede the synthesis and interpretation of evidence in this rapidly evolving field. Assessment methods used to evaluate the effectiveness of ESWT in PF range from functional and pain-related measures to morphological and pathological evaluations, encompassing both subjective and objective outcomes. A clear understanding of the assessment strategies is therefore essential to support both clinical decision-making and future research. In addition to outcome assessment variability, ESWT treatment parameters differ widely across studies, making it difficult for clinicians to gain a comprehensive understanding of optimal treatment protocols. Furthermore, ESWT is frequently compared with diverse conservative interventions across clinical trials, yet the comparative efficacy and application status of ESWT relative to other therapies have not been systematically summarized. To date, no review has comprehensively addressed these interrelated issues. Therefore, this narrative review is structured around the aforementioned domains, providing a detailed overview of three areas (assessment methods; parameters of ESWT; and ESWT versus other interventions) for clinicians and researchers, thereby facilitating understanding and application of ESWT in the management of PF.

## 2. Methods

### 2.1. Databases and Terms

The literature search was conducted in three databases: Web of Science, PubMed, and Cochrane, with the following terms: “shock wave”, “shockwave”, “plantar fascia”, and “plantar fasciitis”. The search strategy is presented in Appendix A. The ZOTERO 7.0.32 software was employed as the management software for search results, in the subsequent steps. The present work was registered in OSF with a DOI of 10.17605/OSF.IO/J2NMZ.

### 2.2. Inclusion and Exclusion Criteria

**Figure 1:**
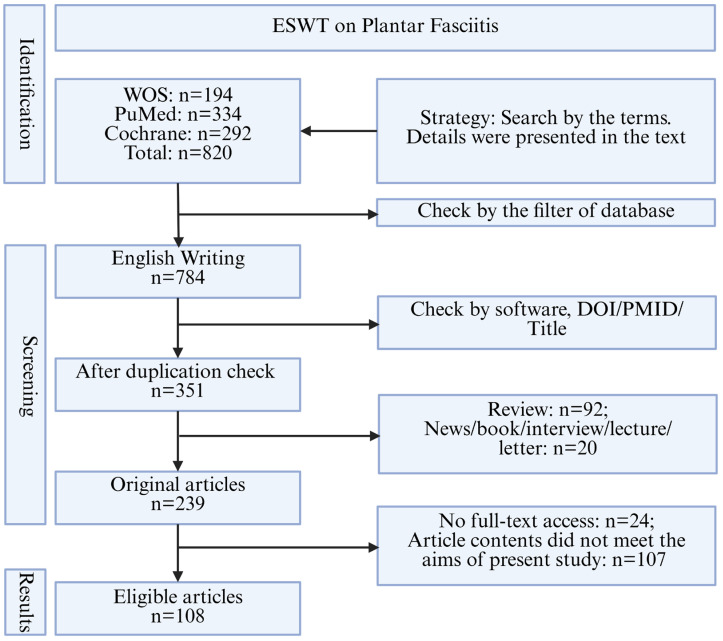
The flowchart of literature searching and screening follows the guidelines of PRISMA.

Based on the above search strategy, studies published up to 30 March 2025, were considered. Inclusion criteria: studies involving human participants diagnosed with PF were eligible, provided that a clear diagnostic criterion for PF was explicitly described in the main text. No restrictions were applied regarding disease severity, symptom duration (acute or chronic), or the presence of calcification; studies containing ESWT intervention were included. Exclusion criteria: studies not written in English; studies of non-original research (e.g., reviews, letters, comments, books, interviews, abstract only, and news); in-vitro studies; studies with no complete assessment method or ESWT parameters; and duplicates. The initial search yielded 820 articles (PubMed: 334, WOS: 194, Cochrane: 292). Of these, 784 were in English, and 351 articles remained after the duplication check. Following the removal of non-original articles, 239 articles remained, including case reports (series), RCTs, non-RCTs, and observational studies. Among the 239 articles, 24 were excluded, due to the inability to obtain the full text. After screening titles, abstracts, and full texts, 108 articles were eligible and were selected for the final analysis. A detailed flowchart of the selection process is shown in Figure 1.

In this study, two authors, Y.S. and Y.W., were responsible for designing and implementing the search strategy, as well as extracting data. Any discrepancies related to inclusion/exclusion decisions or data extraction were resolved through discussion with a third author (C.F.). A predefined data-extraction template was utilized to systematically collect information, including “interventions, prescription of ESWT: pulse/energy/frequency, assessment methods, results, and compared interventions”, to address the main domains (concerns) as described in the Section 1.

## 3. Results

### 3.1. The Assessment Methods Involved in ESWT-PF

**Figure 2:**
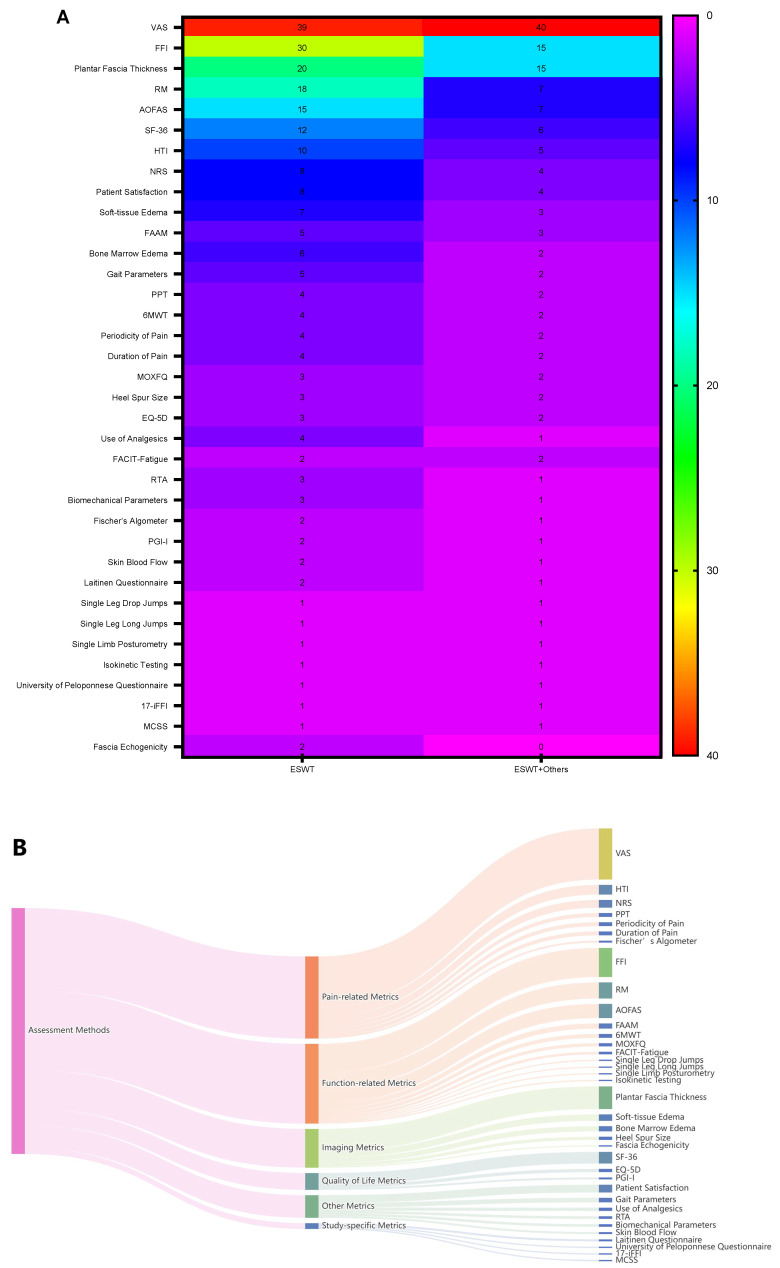

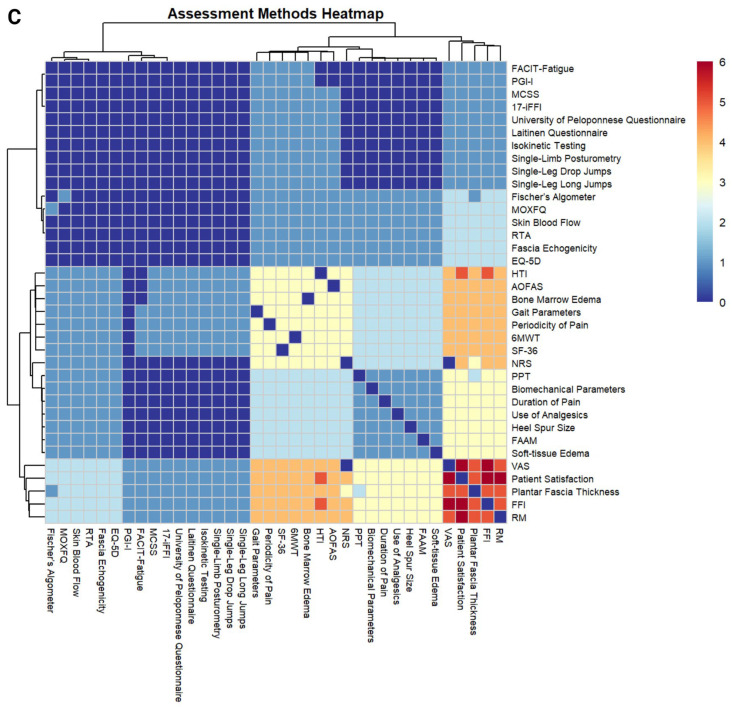
Summary of the assessment methods that were used in ESWT-plantar fasciitis. (**A**) A heatmap of these 36 assessment methods, grouped by ESWT or ESWT+ others. (**B**) A Sankey picture, presenting the categories of these 36 methods. (**C**) A heatmap to present the status of each method combined with another. All abbreviations are presented at the end of the main text.

This section summarizes the assessment methods used in ESWT for plantar fasciitis (PF). All abbreviations are defined and listed at the end of the text. Among the 108 included studies, 51 investigated ESWT alone or versus placebo, while 57 assessed ESWT in combination with, or compared to, other interventions. Assessment methods were classified into six categories: Pain-related metrics (7 methods), Function-related metrics (11 methods), Imaging metrics (5 methods), Quality of Life metrics (3 methods), Other metrics (6 methods), and Study-specific metrics (4 methods). Pain- and function-related metrics were the most frequently used, appearing 127 and 123 times, respectively. In total, 36 distinct assessment methods were identified, with the most common being the Visual Analog Scale (VAS; 79 studies), Foot Function Index (FFI; 45 studies), and plantar fascia thickness (35 studies). Data were visualized separately for ESWT-only studies and ESWT combined with, or compared to, other interventions (Figure 2A,B), and the details of these 36 methods are shown in Figure 2A,B. These findings indicate that pain intensity, functional status, and plantar fascia morphology represent the core domains evaluated in ESWT-related PF studies.

The 36 assessment methods were used either individually or in combination. Common pairings included VAS-FFI, VAS-patient satisfaction, FFI-patient satisfaction, and RM-patient satisfaction following fascia thickness combined with VAS/FFI. A heat map illustrating the relationships among different assessment methods is presented in Figure 2C. Overall, these patterns suggest that multidimensional assessment strategies, integrating pain, functional, and imaging outcomes, are commonly adopted to evaluate treatment effectiveness in PF. Detailed information on all assessment methods is provided in Supplementary Table S1 [13,14,15,16,17,18,19,20,21,22,23,24,25,26,27,28,29,30,31,32,33,34,35,36,37,38,39,40,41,42,43,44,45,46,47,48,49,50,51,52,53,54,55,56,57,58,59,60,61,62,63,64,65,66,67,68,69,70,71,72,73,74,75,76,77,78,79,80,81,82,83,84,85,86,87,88,89,90,91,92,93,94,95,96,97,98,99,100,101,102,103,104,105,106,107,108,109,110,111,112,113,114,115,116,117,118,119,120].

### 3.2. The Overview of ESWT Prescription Parameters

**Figure 3:**
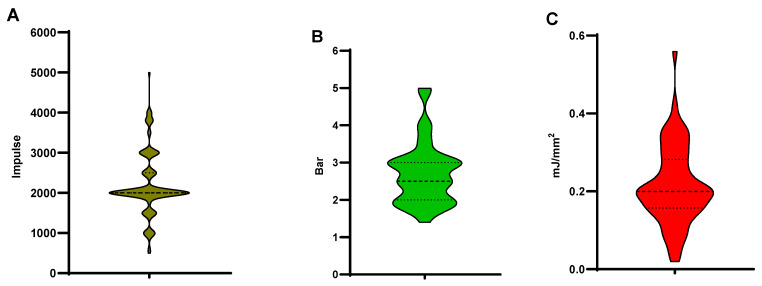
The distribution illustration of ESWT prescription in PF. (**A**) The distribution pattern of impulses; 2000 impulses of one treatment section was the most common one, and was used most frequently in clinical practice, based on all selected 108 studies. (**B**,**C**) The energy density refers to a unit of mJ/mm^2^ or Bar. An intensity of 2.0–3.0 Bar or 0.2 mJ/mm^2^ was the most frequently used energy density, according to all 108 studies.

This section summarizes the prescription parameters of ESWT in studies that included a single ESWT-only intervention group. Considerable variability in treatment protocols was observed across the selected studies. The main prescription parameters extracted included impulses per session, frequency, and energy intensity. The number of impulses per session ranged from 500 to 5000, with 2000 impulses being the most commonly reported value (Figure 3A). Regarding treatment intensity, three types of parameters were used to describe the energy level: air pressure (Bar), energy flux density (mJ/mm^2^), and kilovoltage (kV), although the latter was reported in only a few studies. For air pressure, the reported range was 1.4–5.0 Bar, with 3.0 Bar being the most frequently used intensity, followed by 2.0 and 2.5 Bar. For energy flux density, the most commonly reported values were 0.20 and 0.15 mJ/mm^2^ (Figure 3B,C). Overall, these findings indicate that ESWT prescriptions for PF commonly employ approximately 2000 impulses per session with moderate energy levels, although considerable variability in treatment parameters still exists across studies. Detailed information for all included studies is provided in Supplementary Table S1.

### 3.3. The Relationship of ESWT Prescriptions and Related Outcomes

To explore the relationship between ESWT prescription parameters and clinical outcomes, objective outcome data—including VAS and plantar fascia thickness—were extracted from studies containing an ESWT-only intervention group. Only studies reporting complete pre- and post-treatment mean values were included, while studies presenting partial outcomes or reporting results as ranges were excluded.

After screening the 108 included studies, 69 studies met these criteria and were included in this analysis. Because several studies contained more than one ESWT-only intervention group, the number of datasets exceeded the number of studies.

The extracted outcomes included VAS (*n* = 78), and plantar fascia thickness (*n* = 20). The corresponding prescription parameters collected were impulse count per session and treatment intensity (Bar or mJ/mm^2^). A total of 78 VAS measurements were paired with corresponding shockwave impulse values. Intensity parameters were reported in two units: 38 measurements in mJ/mm^2^ and 24 measurements in bar. Twenty observations of fascial thickness were available. Only data with intensity reported in mJ/mm^2^ (*n* = 10) were included in the present analysis, as a small proportion of the remaining observations reported intensity in bar or kV, which were not directly comparable. Detailed information for all the data in this section is provided in Supplementary Table S2.

**Figure 4:**
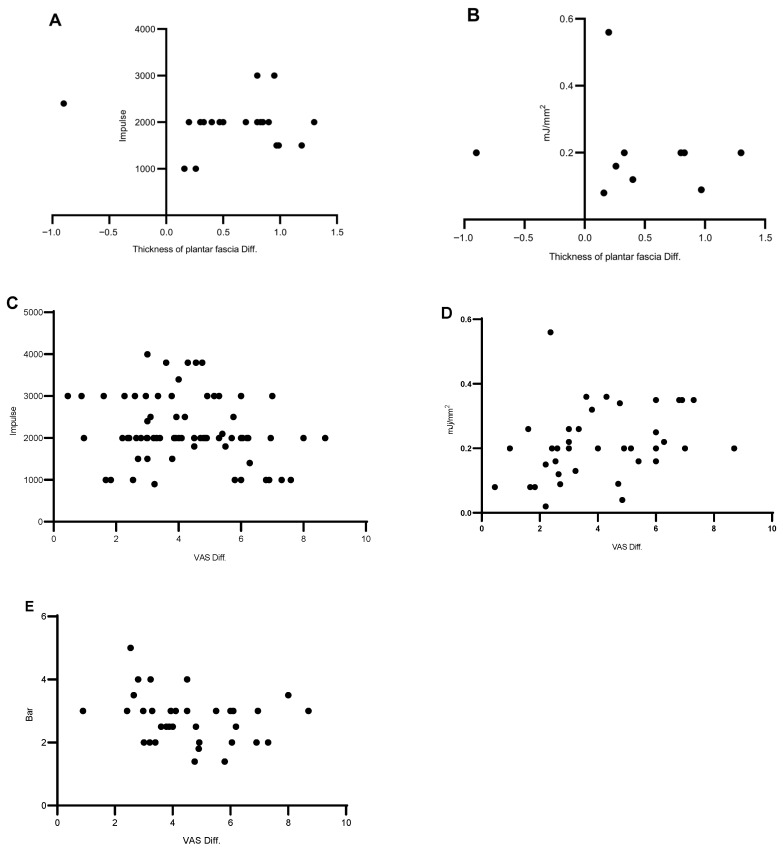
The visualization pictures, prescription parameters, and before/after difference of VAS and plantar fascia thickness. (**A**) The impulses of each session and the thickness difference between the before-treatment and after-treatment results. (**B**) The energy and the thickness difference between the before-treatment and after-treatment results. (**C**–**E**) The impulse/energy and the VAS difference between the before-treatment and after-treatment results. Bar and mJ/mm^2^ are presented separately. Diff: difference. All abbreviations are presented at the end of the main text.

Prescription parameters were visualized, together with the differences between pre- and post-treatment outcomes (Figure 4). Although most studies reported improvements in pain and functional outcomes after ESWT, no consistent relationship between prescription parameters and the magnitude of outcome improvement was observed. These observations are based on heterogeneous clinical trials, and should therefore be interpreted cautiously; they summarize patterns across the available studies, rather than establishing causal relationships between ESWT prescriptions and treatment outcomes.

### 3.4. The Status of ESWT Versus Other PF Interventions

**Figure 5:**
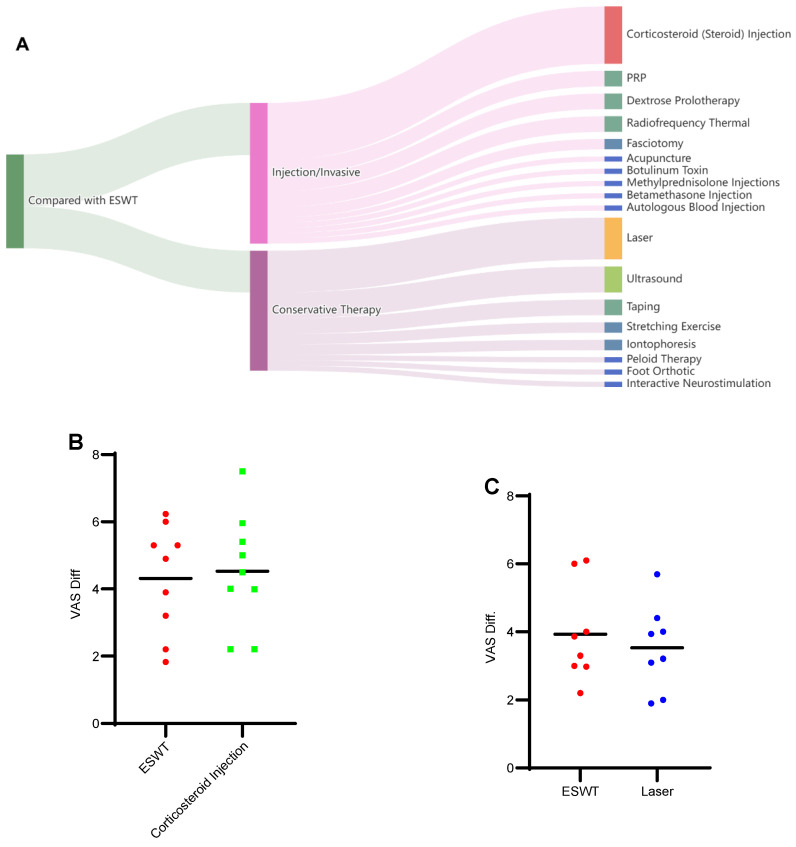
The visualization figures of all ESWT-compared interventions. (**A**) A Sankey picture to present all interventions that are compared to ESWT, divided into two categories (invasive and conservative therapy). (**B**) Data from the studies that focus on ESWT vs. corticosteroid (steroid) injection, the VAS difference of before/after treatment in the ESWT-only group, and corticosteroid injection-only group. (**C**) Data from the studies that focus on ESWT vs. laser, the VAS difference of before/after treatment in the ESWT-only group, and laser-only group. PRP: platelet-rich plasma.

A total of 45 studies met the eligibility criteria for addressing this question, from which outcome data were extracted. Study selection followed the inclusion criteria described in Section 3.3. We first categorized the interventions compared with ESWT and then summarized the comparative outcomes reported across the 45 studies; several studies included multiple intervention arms. As illustrated in Figure 5A, comparator interventions were broadly grouped into two categories: injection/invasive therapies and conservative therapies. Among injection/invasive approaches, corticosteroid injections were the most frequently studied (11 studies), followed by PRP, dextrose prolotherapy, and radiofrequency thermal therapy (three studies each). Laser therapy, ultrasound, taping, and iontophoresis were the most commonly evaluated, with eight, five, three, and two studies, respectively.

#### 3.4.1. Injection/Invasive Therapies

For corticosteroid injections, eleven studies (nine RCTs, one retrospective, and one prospective) were reviewed. Four studies suggested that ESWT was superior, including two reporting a longer duration of action with ESWT [27,30], one showing overall superior effects of ESWT compared with injections [91], and one demonstrating greater improvement in FFI scores with ESWT [65]. Five studies did not demonstrate clear superiority of ESWT: two suggested that injections were more cost-effective and efficacious [39,77], while three reported faster or greater early pain relief with injections [78,93,109]. The remaining two studies reported no overall difference, although injections showed greater short-term benefits and ESWT produced more pronounced long-term pain reduction [54,111].

Overall, corticosteroid injections may provide faster short-term pain relief, whereas ESWT appears to offer more sustained long-term benefits.

PRP: three studies were selected. Two studies [16,90] suggested that PRP is superior to ESWT, while one study [54] demonstrated that neither of these two is superior to the other. Dextrose prolotherapy: the conclusions of three studies point to it seeming to be equal to ESWT. Radiofrequency thermal: two studies suggested that neither is superior, while one study suggested it is superior to ESWT.

#### 3.4.2. Conservative Therapies

For laser therapy, eight studies were involved: three [17,26,40] suggested laser is superior to ESWT, while another three [25,35,61] suggested ESWT is better than laser. The other two [36,84] suggested none is superior to any another.

For ultrasound therapy, four studies [20,21,84,114] showed the superiority of ESWT, and that ESWT is faster or more efficient. One study [45] suggested that ultrasound has a great effect on FFI, and that the ESWT has its own advantage in proprioception.

Regarding taping, one study reported significant functional improvement after six months with ESWT, whereas another found comparable effects between ESWT and taping; a third suggested superior functional gains with taping. For iontophoresis [46,66], one study observed short-term benefits with ESWT, while another reported no clear superiority between the interventions. Data on additional interventions are provided in Supplementary Table S3; interventions supported by only a small number of studies are not discussed in detail here.

The difference for VAS before/after treatment was extracted and calculated from those studies that focus on corticosteroid (steroid) injection and laser (other outcomes were lacking enough studies and data), compared to the VAS difference of the ESWT-group (as presented in Figure 5B). Two studies on corticosteroid injections did not report VAS data. Overall, current data are insufficient to determine a clear advantage of either treatment. Detailed information for this analysis is provided in Supplementary Table S3.

### 3.5. Characteristics of the Study Populations

**Figure 6:**
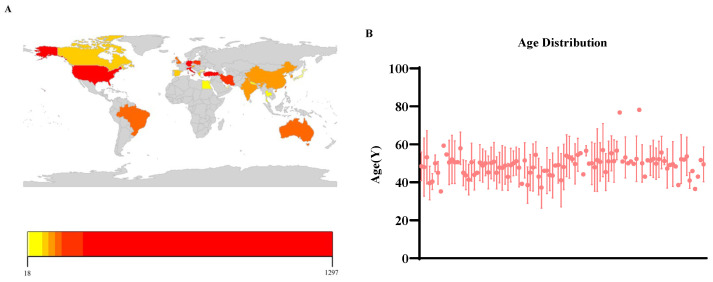
Demographic characteristics of populations across the 108 included studies. (**A**) Geographic distribution of ESWT-treated patient populations. The number of patients per country ranged from 18 to 1297. (**B**) Age distribution of patients across all eligible studies. Age data are reported as mean ± standard deviation or mean where available.

The characteristics of study populations across the selected studies were heterogeneous. A total of 108 studies were conducted in 24 countries. Among these, Turkey, Germany, the United States, and Italy contributed the largest number of studies, with 31, 11, 9, and 8 studies, respectively (Figure 6A; Supplementary Table S4). Regarding the patient populations, Turkey, Germany, and the United States included the largest numbers of patients who received ESWT therapy, with 1297, 712, and 612 individuals, respectively. It should be noted that these figures represent only patients treated with ESWT, not the total patient population across all 108 studies. Overall, 4781 patients received ESWT therapy in these studies. Seventeen studies did not report patients’ gender, while the remaining 91 studies provided complete gender information, comprising 2623 females and 1415 males. These results indicate that plantar fasciitis (PF) is more prevalent in females than in males, consistent with previous findings by Thompson et al. [5]. Age distribution data are presented in Figure 6B. Where available, age was reported as mean ± standard deviation or as mean; in some studies, only median values were provided, and not all studies reported age. Among the studies reporting age, most patients were between 40 and 60 years old, a distribution pattern that aligns with prior observations [5].

## 4. Discussion

The present review summarizes the current status of ESWT for plantar fasciitis by integrating evidence on assessment methodologies, treatment parameters, and comparative strategies. This comprehensive perspective is intended to support clinicians in efficiently understanding ESWT and making informed clinical decisions. In contrast to previous reviews that primarily emphasized comparative efficacy between ESWT and other conservative treatments to inform evidence-based practice, this work provides a detailed, practice-oriented overview of ESWT application, enabling clinicians—particularly those with limited experience—to rapidly grasp its scope and implementation in routine care.

First, the findings of this review indicate that assessment methodologies for plantar fasciitis are diverse and encompass multiple domains, including pain, function, morphology (plantar fascia thickness), quality of life, and other condition-specific measures. These tools range from subjective to objective assessments, and collectively reflect the primary symptoms and clinical concerns of affected patients; however, no single method is sufficient to capture all relevant manifestations, underscoring the need for combined assessment approaches. Each assessment tool serves distinct purposes in both clinical and research settings, suggesting that method selection should be tailored to individual patient characteristics and study objectives. The use of an optimized set of complementary assessment methods may improve the accuracy of treatment evaluation and support individualized therapeutic strategies. With respect to treatment prescription, this review highlights key ESWT parameters, particularly energy flux density and the number of impulses. As detailed in Section 3.2, the reported parameter ranges were relatively concentrated, indicating a limited but commonly adopted prescription profile. These findings may assist clinicians with limited experience in ESWT to make informed parameter selections efficiently and to implement integrated treatment and evaluation protocols in conjunction with appropriate assessment methods. This constitutes a primary objective and central contribution of the present review.

Second, data from 79 studies were synthesized to calculate pre- and post-treatment changes in VAS, FFI, HTI, and plantar fascia thickness, providing an initial overview of the relationship between ESWT prescription parameters and treatment effects (Section 3.3). However, owing to the narrative design of the present review, these findings do not constitute sufficient evidence to establish a direct causal relationship. Moreover, ESWT outcomes are influenced by the combined effects of energy flux density and impulse number, precluding definitive recommendations regarding optimal prescription parameters. Further high-quality, evidence-based studies are therefore required to clarify parameter–outcome relationships and to inform clinical optimization.

Third, the comparative landscape of ESWT relative to other interventions was preliminarily synthesized. Comparator treatments broadly comprised two categories: invasive and non-invasive interventions, both of which demonstrated variable outcomes relative to ESWT, reflecting a lack of consensus across existing studies. Corticosteroid injection, the most frequently investigated invasive intervention, exhibited particularly heterogeneous comparative effects. Some studies reported that ESWT provided longer-lasting benefits and superior functional improvement, particularly in FFI scores, while both treatments were effective in pain reduction [27,30,65]. In contrast, other studies suggested that corticosteroid injection was more cost-effective and efficacious, superior to ESWT, or that neither intervention demonstrated clear superiority.

One critical but often overlooked factor contributing to these inconsistencies is the heterogeneity of ESWT prescription parameters. Across studies comparing ESWT with corticosteroid injection (*n* = 11), substantial variability was observed, particularly in energy flux density, which ranged from 0.15 to 0.29 mJ/mm^2^. Similar prescription heterogeneity was also present in studies comparing ESWT with other interventions. In addition, assessment methodologies varied considerably among studies, and differences in outcome measures may have contributed to divergent conclusions. The timing of outcome evaluation was also inconsistent, further complicating cross-study comparisons.

Beyond methodological variability, fundamental differences in therapeutic mechanisms may also account for the observed discrepancies. Corticosteroid injections exert anti-inflammatory effects primarily through suppression of inflammatory mediators; however, they may also inhibit fibroblast proliferation and extracellular matrix synthesis, potentially compromising tissue biomechanical integrity [6,121]. By contrast, ESWT and other non-invasive modalities modulate inflammation through alterations in the physicochemical environment of tissues and cells [8], with ESWT demonstrating additional regenerative effects on soft tissue [122]. Physical exercise interventions primarily aim to enhance biomechanical function and physical performance. Collectively, these mechanistic differences may partly explain the divergent outcomes observed between ESWT and alternative treatments, across specific clinical domains.

The effectiveness of ESWT is influenced not only by prescription parameters, but also by the selection of the treatment area. In plantar fasciitis, treatment is most commonly applied to local trigger points chosen by the operator. However, one study [108] suggested that targeting a combined area—including both local and remote sites, guided by fascia manipulation theory—can enhance efficacy and achieve notable clinical outcomes. Clinically, this finding indicates that treatment-area selection should not be considered a fixed or secondary factor, but rather a potentially modifiable and critical component of ESWT prescription. Awareness of alternative treatment-area strategies may help clinicians move beyond operator-dependent local tender-point selection, and adopt approaches informed by underlying mechanisms. This also underscores the need for standardized reporting and clear definitions of treatment areas in future studies.

This review has several limitations. First, some relevant studies may have been missed, due to the search strategy. Second, the narrative design and heterogeneity of included studies precluded a formal risk-of-bias assessment, which may introduce potential bias into the conclusions. High-quality systematic reviews and meta-analyses are needed, to provide more definitive evidence.

## 5. Conclusions

This review provides a comprehensive analysis of the current status of ESWT in the treatment of PF. First, 36 distinct assessment methods were obtained from 108 studies, and were classified into six categories, including pain (seven methods, with the Visual Analog Scale being the most frequently used), function (eleven methods, most commonly the Foot Function Index), Imaging metrics (five methods, with most common being plantar fascia thickness by ultrasound), Quality of Life metrics (three methods), Other metrics (six methods), and Study-specific metrics (four methods). Second, the review systematically categorizes the diverse prescription parameters, such as impulse count, energy flux density, and frequency, which differ across studies. The most commonly applied prescription parameters are 2000 impulses per session and an energy flux density of 0.2 mJ/mm^2^ or 3.0 bar. Third, the comparison status of ESWT relative to other interventions was examined. Across 18 comparative treatments, corticosteroid injections, PRP, dextrose prolotherapy, laser therapy, and ultrasound were the most frequently compared modalities. ESWT and comparator interventions demonstrated differential advantages across specific outcomes; there is no clear evidence supporting the superiority of one approach over the other. These variabilities underscore the importance of individualized treatment protocols tailored to specific patient needs.

## Data Availability

No new data were created or analyzed in this study.

## Supplementary Materials

The following supporting information can be downloaded at: https://www.mdpi.com/article/10.3390/jfmk11010123/s1.

## Abbreviations

Abbreviation	Full spelling
VAS	Visual Analog Scale
NRS	Numerical Rating Scale
PPT	Pain Pressure Threshold
HTI	Heel Tenderness Index
FFI	Foot Function Index
AOFAS	American Orthopedic Foot and Ankle Society Score
RM	Roles and Maudsley Score
FAAM	Foot and Ankle Ability Measure
MOXFQ	Manchester–Oxford Foot Questionnaire
FACIT	Functional Assessment of Chronic Illness Therapy-Fatigue Scale
6MWT	6-Minute Walking Test
SF-36	Short Form-36
PGI-1	Patient Global Impression of Improvement
RTA	Return to Activity
University of Peloponnese Questionnaire	University of Peloponnese Pain, Functionality, and Quality of Life Questionnaire
17-Iffi	17-Italian Foot Function Index
MCSS	Mayo Clinical Scoring System

## Appendix A

The search strategies were tailored to each database as follows: PubMed: “(shock wave [Title/Abstract] or shockwave [Title/Abstract]) AND (plantar fascia [Title/Abstract] OR plantar fasciitis [Title/Abstract])”; WOS: (“shockwave” or “shock wave” [Title]) and (“plantar fasciitis” or “plantar fascia” [Title]); Cochrane: (“shockwave” or “shock wave” [Title Abstract Keyword]) and (“plantar fascia” or “plantar fasciitis” [Title Abstract Keyword]).
