# Supplementary material for: A Narrative Review of Shockwave Therapy in Plantar Fasciitis
Source: J Funct Morphol Kinesiol. 2026 Mar 17;11(1):123. doi: 10.3390/jfmk11010123 (PMC13028312; doi:10.3390/jfmk11010123)
Supplement: Supplementary file 1 [file jfmk-11-00123-s001.zip › Table S4.pdf]

Table S4 The Basic Information of All Selected Studies

Nation	Quantity of Study	Quantity of Patient
Turkey	31	1297
Germany	11	712
US	9	612
Italy	8	266
China	2	130
Iran	6	162
Singapore	1	19
Greece	1	88
Spain	2	102
Thailand	2	31
China Taiwan	5	206
Japan	1	23
Nepal	1	30
Canada	2	83
Korea	3	119
Poland	5	167
Brazil	4	158
Australia	2	141
Austria	2	104
Egypt	3	73
UAE	1	18
UK	2	98
Bulgaria	1	21
India	3	121
